# The confusion assessment method for the intensive care unit (CAM-ICU) and intensive care delirium screening checklist (ICDSC) for the diagnosis of delirium: a systematic review and meta-analysis of clinical studies

**DOI:** 10.1186/cc11407

**Published:** 2012-07-03

**Authors:** Dimitri Gusmao-Flores, Jorge Ibrain Figueira Salluh, Ricardo Ávila Chalhub, Lucas C Quarantini

**Affiliations:** 1 Intensive Care Unit, University Hospital Prof. Edgar Santos, Universidade Federal da Bahia, Rua Augusto Viana, Salvador, 40110-910, Brazil; 2 Programa de Pós-graduação em Processos Interativos dos Órgãos e Sistemas, Instituto de Ciências da Saúde (ICS), Universidade Federal da Bahia, Av. Reitor Miguel Calmon, Salvador, 40110-902, Brazil; 3 D'Or Institute of Research and Education, Rua Diniz Cordeiro, 30, Rio de Janeiro, 22281-100, Brazil; 4 PostGraduate Program, Instituto Nacional do Câncer (INCA), Praça Cruz Vermelha, 23, Rio de Janeiro, 20230-130, Brazil; 5 Department of Neurosciences and Mental Health, University Hospital Prof. Edgar Santos, Universidade Federal da Bahia, Rua Augusto Viana, Salvador, 40110-910, Brazil; 6 Programa de Pós-graduação em Medicina e Saúde (PPgMS) - Faculdade de Medicina da Bahia, Universidade Federal da Bahia, Rua Augusto Viana, Salvador, 40110-910, Brazil

## Abstract

**Introduction:**

Delirium is a frequent form of acute brain dysfunction in critically ill patients, and several detection tools for it have been developed for use in the Intensive Care Unit (ICU). The objective of this study is to evaluate the current evidence on the accuracy of the Confusion Assessment Method for Intensive Care Unit (CAM-ICU) and the Intensive Care Delirium Screening Checklist (ICDSC) for the diagnosis of delirium in critically ill patients.

**Methods:**

A systematic review was conducted to identify articles on the evaluation of the CAM-ICU and the ICDSC in ICU patients. A MEDLINE, SciELO, CINAHL and EMBASE databases search was performed for articles published in the English language, involving adult populations and comparing these diagnostic tools with the gold standard, the Diagnostic and Statistical Manual of Mental Disorders (DSM-IV) criteria. Results were summarized by meta-analysis. The QUADAS scale was used to assess the quality of the studies.

**Results:**

Nine studies evaluating the CAM-ICU (including 969 patients) and four evaluating the ICDSC (*n *= 361 patients) were included in the final analysis. The pooled sensitivity of the CAM-ICU was 80.0% (95% confidence interval (CI): 77.1 to 82.6%), and the pooled specificity was 95.9% (95% CI: 94.8 to 96.8%). The diagnostic odds ratio was 103.2 (95% CI: 39.6 to 268.8). The pooled area under the summary receiver operating characteristic curve (AUC) was 0.97. The pooled sensitivity of the ICDSC was 74% (95% CI: 65.3 to 81.5%), and the pooled specificity was 81.9% (95% CI: 76.7 to 86.4%). The diagnostic odds ratio was 21.5 (95% CI: 8.51 to 54.4). The AUC was 0.89.

**Conclusions:**

The CAM-ICU is an excellent diagnostic tool in critically ill ICU patients, whereas the ICDSC has moderate sensitivity and good specificity. The available data suggest that both CAM-ICU and the ICDSC can be used as a screening tool for the diagnosis of delirium in critically ill patients.

## Introduction

Delirium is a prevalent form of acute brain dysfunction that occurs in critically ill patients [1]. Despite its elevated frequency and association with increased morbidity and mortality [2], delirium remains an underdiagnosed condition in the intensive care unit (ICU), and a standard clinical evaluation does not have an adequate accuracy for the diagnosis [3]. Several methods have been developed and validated to diagnose delirium in ICU patients [4], but the Confusion Assessment Method for the Intensive Care Unit (CAM-ICU) and the Intensive Care Delirium Screening Checklist (ICDSC) are the most frequently employed tools for this purpose [5].

Since the validation of the CAM-ICU and the ICDSC, these tools have been translated into and validated in many languages [6-12] and have been widely employed in clinical practice [5,13]. However, studies show different results regarding their accuracy for the diagnosis of delirium, possibly affecting the reported incidence of this clinical condition and the implementation of prompt preventive and therapeutic measures [14].

The aim of this study was to perform a systematic review and use a meta-analytic approach to pool previously published studies presenting data on the CAM-ICU and the ICDSC for the diagnosis of delirium in the critically ill.

## Materials and methods

**Figure 1:**
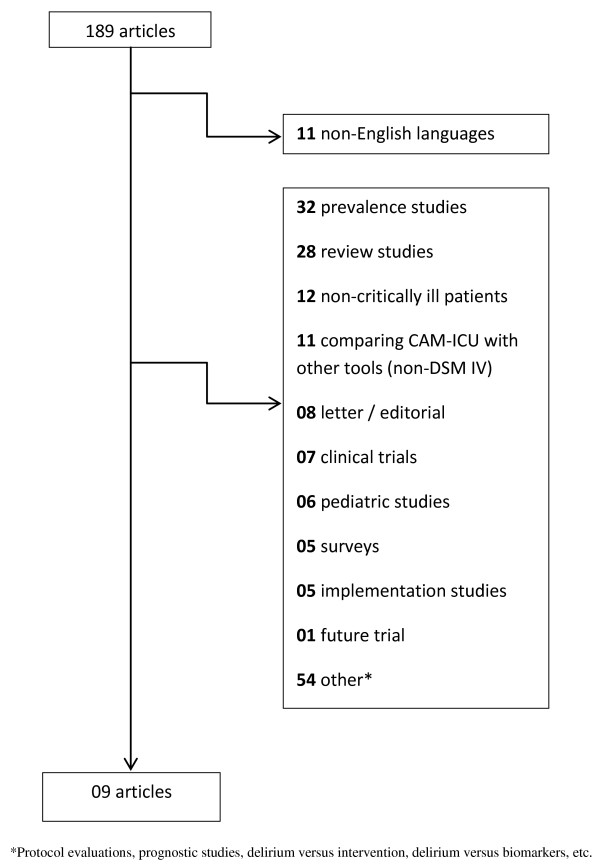
**Flow diagram of the literature search for studies evaluating the CAM-ICU performance**.

**Figure 2:**
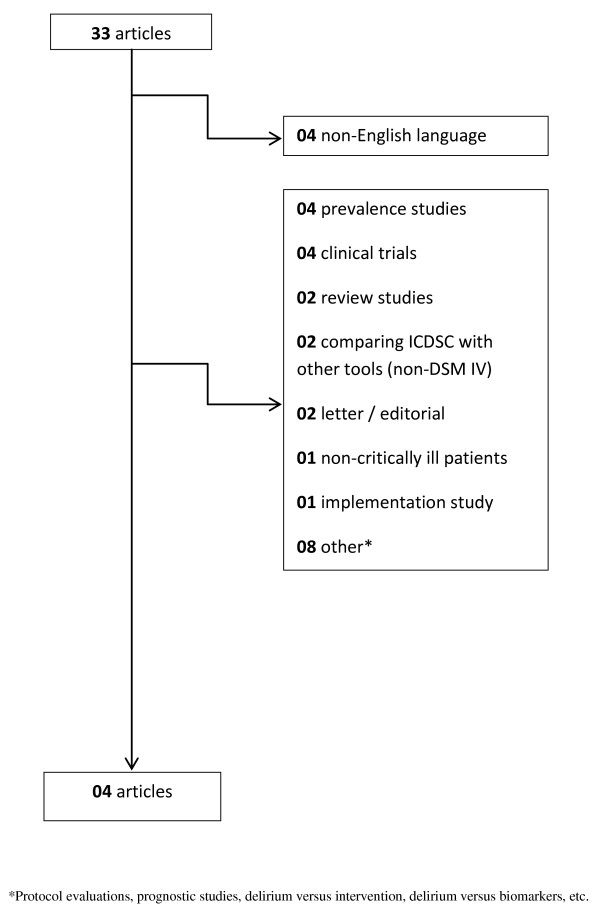
**Flow diagram of the literature search for studies evaluating the ICDSC performance**.

A two-stage systematic review process was performed. First, we performed a systematic MEDLINE, SciELO, CINAHL and EMBASE databases search using the keywords "CAM-ICU" or "Confusion Assessment Methods for the Intensive Care Unit" (Figure 1) and "ICDSC" or "Intensive Care Delirium Screening Checklist" (Figure 2) from January 2001 through 18 November 2011. However, the automatic alert system of MEDLINE was used to identify studies published during the process of the analysis of the results.

The aim of the review was to identify full-text, English-language publications that evaluated the performance of the CAM-ICU and the ICDSC in critically ill patients. Only articles comparing these diagnostic tools with the gold standard, the DSM-IV criteria, were included.

Original peer-reviewed studies involving the adult population were selected and analyzed. We excluded case reports, review articles, studies that have used these tools to evaluate the correlation between delirium and morbidity or mortality, or compare it with other tools (those not based on DSM-IV criteria). All letters and comments were analyzed for information on validation or implementation of these tools. Studies that assessed children were initially excluded, but further analysis was performed and data including this distinct population were attached in the Additional file 1, Figures S1 and S2. In stage two, eligibility assessment (articles comparing the CAM-ICU or the ICDSC with DSM-IV criteria) and data abstraction were performed independently in an unblinded, standardized manner by two reviewers (DGF and RAC). Discrepancies in the search were resolved by consensus among the authors.

Subsequently, the identified articles were screened electronically. For each eligible article, using a predefined categorization system, information was extracted on the authors, journal, year of publication, study design, inclusion period, number of patients, number of observations, patient population, total number of patients diagnosed with delirium, APACHE II score and sensitivity and specificity of the CAM-ICU and the ICDSC. Moreover, when available, we extracted information about accuracy of these tools in different subgroups of patients analyzed in each paper.

In studies that involved more than one assessor (nurses and/or physicians) in the process of the validation of the tools, we selected the highest sensitivity and included it in the meta-analysis (data from all evaluators are attached in Additional file 2, Table S1). In addition, several studies evaluated the same patient at multiple time-points with different diagnostic tools. In this case, the accuracy of the tool was calculated based on the total number of assessments and not on the number of patients.

The QUADAS scale (first version) was employed to assess the quality of the studies [15]. This tool was developed specifically to assess the quality of studies of diagnostic accuracy included in systematic reviews. Fourteen items were evaluated and, accordingly, each included study was scored from 0 to 14, with a high value indicating a better quality of study. Finally, results were summarized by meta-analysis.

### Statistical analysis

All of the tests were performed using the package STATA v. 9.0 and MetaDiSC^® ^(Unit of Clinical Biostatistics Team of the Ramón y Cajal Hospital, Madrid, Spain) [16] adopting a significance level of 0.05.

The MetaDiSC^® ^software was used to calculate the pooled values of sensitivity, specificity and diagnostic odds ratios of each of the tools. The heterogeneity of the studies was checked by the chi-square test (*P *≤0.05). The summary receiver operating curve characteristic (SROC) was also drawn. In the SROC graph, each point comes from a different study. The area under the curve (AUC) reflects the overall performance of the test. The heterogeneity between studies was analyzed with chi-square statistics.

## Results

Nine studies evaluating the CAM-ICU and four evaluating the ICDSC were included in the final analysis. Of these, two studies validated both tools simultaneously [9,17]. The main characteristics of the studies are depicted in Tables 1 and 2.

**Table 1:** Main characteristics of the included studies (evaluation of the CAM-ICU)

Author	N	Year	ICU	Language	Delirium N (%)	Sensitivity	Specificity	APACHE II	QUADAS
**Ely **[19]	38	2001	Medical Coronary	English	33 (87)^1^	100 (95.2 to 100)	100 (79.4 to 100)	17.1 ± 8.7^#^	13
**Ely **[18]	96	2001	Medical Coronary	English	(25.2)^2^	100 (95.4 to 100)	88.8 (83.8 to 92.7)	23 (18 to 29)*	13
**Lin **[7]	102	2004	Medical	Chinese	22 (22.4)	95.5 (77.2 to 99.9)	97.5 (91.3 to 99.7)	NR	13
**van Eijk **[17]	126	2009	General	English Dutch	43 (34)	64.3 (48.0 to 78.4)	88.8 (79.0 to 94.1)	20. 9 ± 7.5^#^	14
**Luetz **[4]	156	2010	Surgical	German	63 (40)	78.8 (72.0 to 84.5)	97.1 (94.9 to 98.5)	16 (13 to 19)*	14
**Heo **[11]	22	2011	Medical	Korean	16 (72.7)	89.5 (78.5 to 98.0)	71.4 (47.8 to 88.7)	25.5 (9 to 39)*	13
**van Eijk **[14]	181	2011	Medical Surgical	English Dutch	80 (28.3)	46.7 (35.1 to 58.6)	98.1 (93.4 to 99.8)	18.6 ± 7.5^#^	13
**Gusmao-Flores **[9]	119	2011	Medical Surgical	Portuguese	46 (38.6)	72.5 (56.1 to 85.4)	96.2 (89.3 to 99.2)	15 ± 6^#^	14
**Mitasova **[10]	129	2012	Stroke Unit	Czech	55 (42.6)^1^	78.9 (73.7 to 83.5)	98.3 (97.1 to 99.1)	NR	14

^1^During hospitalization, 2% of daily evaluations, NR, not reported, ^# ^mean, * median.

**Table 2:** Main characteristics of the included studies (evaluation of the ICDSC)

Author	N	Year	ICU	Language	Delirium N (%)	Sensitivity	Specificity	APACHE II	QUADAS
**Bergeron **[20]	93	2001	Medical Surgical	English	15 (16%)	93.3 (68.1 to 99.8)	80.8 (70.3 to 80.8)	14 (8 to 21)*	13
**Van Eijk **[17]	126	2009	Medical Surgical	English Dutch	43 (34)	42.9 (27.7 to 59.0)	94.7 (87.1 to 98.5)	20.9 ± 7.5^#^	13
**George **[21]	59	2011	Medical Cardiac	English	20 (33.9)	75.0 (50.9 to 91.3)	74.4 (57.9 to 87.0)	NR	13
**Gusmao-Flores **[9]	119	2011	Medical Surgical	Portuguese	46 (38.6)	95.7 (85.2 to 99.5)	72.6 (60.9 to 82.4)	15 ± 6^#^	13

NR: not reported, ^# ^mean, * median

A total of 969 patients were included for the evaluation of the CAM-ICU in the nine studies identified, whereas 391 patients were evaluated in the four validation studies of the ICDSC. All of the studies were conducted in the ICU and, except for the study of van Eijk *et al. *[14], all of the studies used a methodology for the validation of diagnostic tools. The study by van Eijk *et al. *[14] evaluated the CAM-ICU in daily practice.

### Studies assessing the CAM-ICU

Of the nine studies evaluating the CAM-ICU, only two were multicenter evaluations [9,14]. A mixed population of critically ill patients was evaluated. Two studies exclusively evaluated patients on mechanical ventilation [7,18], while the other studies evaluated ventilated and non-ventilated patients [4,9,11,14,17,19], and one study exclusively evaluated stroke patients regardless of the ventilatory status [10].

Only the first validation study of the CAM-ICU [19] did not use the sedation scale RASS (Richmond Agitation Sedation Scale), so feature 4 of the CAM-ICU was considered to be positive when the patient presented with an altered level of consciousness (other than alert). In the studies using RASS, patients were excluded if RASS < -3. Only the study by Luetz *et al. *excluded patients with RASS ≤-3 [4].

The accuracy of the CAM-ICU was evaluated in subgroups of patients with RASS < 0 in two studies [4,10]. In a population of patients with stroke, the sensitivity of the CAM-ICU was higher in this subgroup (85% versus 78.9%) [10], and a similar finding was observed in surgical patients (85% versus 78.8%) [4].

The median quality (QUADAS) score was 13 (range 13 to 14). Studies that received a score of 13 were not scored on item 4 of this tool [7,11,14,18]; that is, they did not mention or spent a long time between the application of the CAM-ICU and DSM-IV criteria.

Five studies classified the subtypes of delirium (hypoactive, hyperactive and mixed) [7,9,10,14,17]. In two studies, the accuracy of the CAM-ICU was evaluated in these patient subgroups [14,17], and a lower sensitivity was observed in hypoactive delirium. van Eijk *et al. *showed an overall sensitivity of the CAM-ICU of 64.3% and only of 57% in patients with hypoactive delirium [17]. The same group of researchers, in a multicenter study, showed only 31% sensitivity by the CAM-ICU in these subtypes of delirium, whereas the global sensitivity they obtained was 46.7% [14].

No studies compared the accuracy of the CAM-ICU in ventilated versus non-ventilated patients. However, Ely *et al. *[19] evaluated a subgroup of patients undergoing mechanical ventilation (*n *= 22 patients) and found a slight increase in the sensitivity (100%) and a slight decrease in the specificity (88%) compared with the overall sample.

Gusmao-Flores *et al. *described 11 evaluations in patients with noninvasive ventilation [9]. These authors reported excellent accuracy for the CAM-ICU: 100% for both sensitivity and specificity.

### Studies assessing the ICDSC

Four studies with mixed populations evaluated the ICDSC, only one of which was a multicenter study [9].

All studies that evaluated the ICDSC used the tool as the original study [20]. The ICDSC was rated by the evaluator based on the patient's reports of the previous 24 hours, using a cutoff of 4. Due to these features, the quality (QUADAS) score was 13 for all of the studies.

Two studies suggest an improved accuracy with different cutoffs [9,21]. George *et al. *showed an optimal threshold for screening with a score of 3 [21]. Compared with a cutoff of 4, the sensitivity increased from 75% to 90%; however, the specificity decreased from 74.3% to 61.5%. After excluding cases that were considered to be subsyndromal delirium (a cutoff of 3), Gusmao-Flores *et al. *suggested a better specificity with a cutoff of 5, which identified correctly 86.5% of cases [9].

Only one study evaluated the accuracy of the ICDSC in different subtypes of delirium [17] and this tool presented a lower sensitivity in hypoactive delirium (42.9% versus 32%).

### Meta-analysis of studies assessing the CAM-ICU

**Figure 3:**
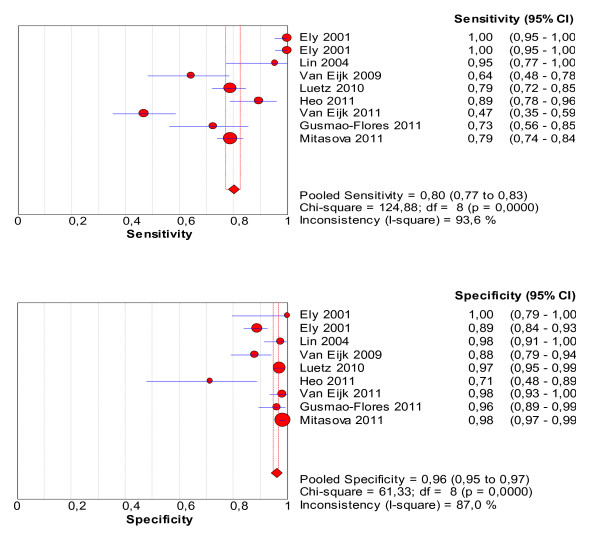
**Forest plot of the pooled values of sensitivity and specificity of the CAM-ICU**.

**Figure 4:**
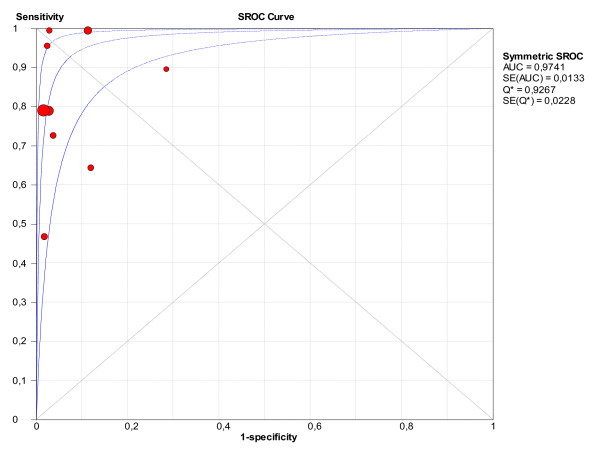
**Summary receiver operating characteristics (SROC) obtained from the evaluation studies of the CAM-ICU**.

The pooled values of sensitivity and specificity for the CAM-ICU were 80.0% (95% confidence intervals (CI): 77.1 to 82.6%) and 95.9% (95% CI: 94.8 to 96.8%), respectively (Figure 3). The area under the SROC was 0.97 (Figure 4), suggesting excellent accuracy.

### Meta-analysis of studies assessing the ICDSC

**Figure 5:**
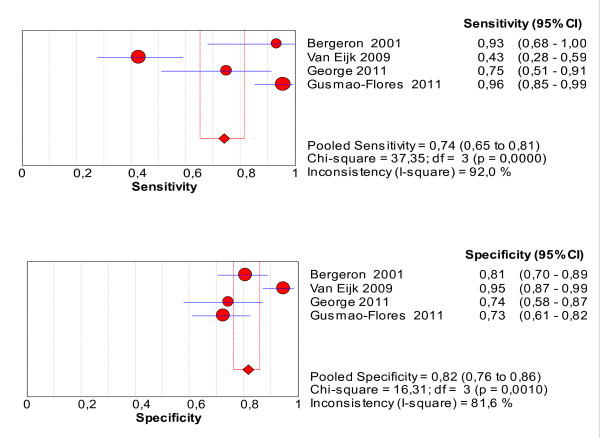
**Forest plot of the pooled values of sensitivity and specificity of the ICDSC**.

**Figure 6:**
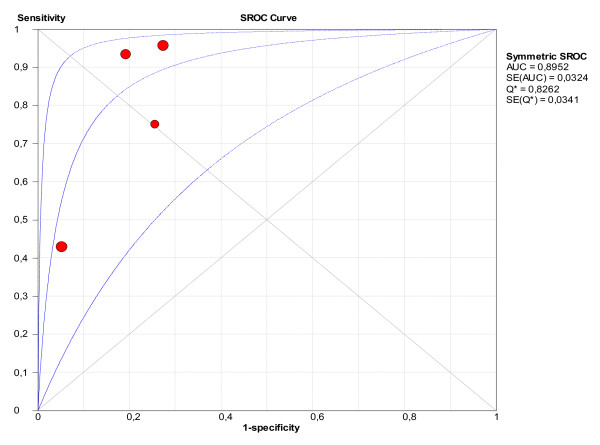
**Summary receiver operating characteristics (SROC) obtained from the evaluation studies of ICDSC**.

The pooled values of sensitivity and specificity for the ICDSC were 74% (95% CI: 65.3 to 81.5%) and 81.9% (95% CI: 76.7 to 86.4%), respectively (Figure 5). The area under the SROC was 0.89 (Figure 6), suggesting good accuracy.

## Discussion

This study represents the first attempt to synthesize the validity and added value of the CAM-ICU and ICDSC in ICU patients. Our results showed that the overall accuracy of the CAM-ICU is excellent, with pooled values for sensitivity and specificity of 80% and 95.9%, respectively. In addition, the pooled values for the sensitivity and specificity of the ICDSC were 74% and 81.9%, respectively. Thus, the currently available data support the use of the CAM-ICU or of the ICDSC as screening tools for delirium in critically ill patients. In addition, because of its high specificity, the CAM-ICU is an excellent diagnostic tool to delirium. This is relevant because a validated tool should be used routinely for monitoring critically ill patients and when delirium is present an algorithm to investigate its cause and a therapeutic strategy should be performed.

After the first validation study [19], the CAM-ICU was translated into and validated in many languages [6-9,11,22]. Although studies published in non-English languages have been excluded from this systematic review and meta-analysis, some have shown similar accuracy to the CAM-ICU. Tobar *et al. *evaluated 29 ventilated patients in the ICU and showed a sensitivity and specificity of 80% and 96%, respectively [6]. Additionally, Toro *et al. *evaluated 129 patients and observed a sensitivity of 79.4% and a specificity of 97.9% for the CAM-ICU [8]. These same authors performed a subgroup analysis with the ventilated patients (*n *= 29), and the results suggested better sensitivity (92.9% versus 79.4%) and worse specificity (86.7% versus 97.9%) in this subgroup of patients. Both studies were published in the Spanish language. Chuang *et al. *validated a Chinese version of the CAM-ICU and again reported high sensitivity (96%) when it was performed by a physician [12].

The present meta-analysis has shown that the pooled sensitivity of the CAM-ICU was 80%, which demonstrates that this tool has good performance for screening patients with delirium in ICU. However, it is also evident that no other validation study has found as high a sensitivity as was observed in the initial studies by Ely *et al. *[18,19]. In addition, there was an even lower sensitivity when the CAM-ICU was used in daily practice, that is, outside of a methodology for validation [14].

Although there is no clear explanation for this loss of sensitivity in the most recent studies, it is possible that the evaluation in cohorts of patients that were less sedated, which is a current trend [23], contributes to decreases in the accuracy of the CAM-ICU. In this systematic review, a higher sensitivity of the CAM-ICU was observed in two studies in subgroups of patients with RASS < 0 [4,10]. Also, a higher sensitivity seems to be present in sedated patients and it is suggested by the differences in accuracy of the CAM-ICU between ventilated and non-ventilated patients. Although no studies compared the accuracy in these subgroups of patients, the study by Toro *et al. *[8] (not included in this systematic review) is consistent with Ely's study [19] and indicates excellent sensitivity in the subgroup of patients undergoing mechanical ventilation. Again, perhaps the sedation effects can contribute to these findings. It is reasonable to hypothesize that feature 2 (inattention) or feature 3 (disorganized thinking) of the CAM-ICU is less likely to be detected when patients are less sedated. Recently, Vasilevskis *et al. *suggested a more intense approach to the detection of inattention when the CAM-ICU is used in daily practice [24]. In addition, feature 1 (an acute onset of mental status changes) may be most frequently considered to be positive in patients with sedation and thus increases the sensitivity of the tool. Of course, more studies are necessary to explain and prove this hypothesis.

The four features of the CAM-ICU - 1) acute onset of mental status changes or fluctuating course; 2) inattention; 3) disorganized thinking; and 4) altered level of consciousness - have objective definitions. This characteristic likely justifies the high inter-rater reliability reported in several studies [4,10,11,18,19].

Moreover, the specificity of the CAM-ICU is high. The pooled value for specificity was 96%, suggesting that when the CAM-ICU is positive, it is not necessary to confirm the diagnosis of delirium by the DSM-IV criteria, improving its feasibility in the ICU. In other words, the CAM-ICU is not only adequate for screening but also a good confirmatory diagnostic tool for delirium in critically ill patients.

Recently, Guenther *et al. *published a study of the accuracy of the CAM-ICU Flowsheet, comparing it with the DSM-IV criteria [25]. Interestingly, they found a sensitivity of 88% to 92% and an excellent specificity of 100%. Clearly, the CAM-ICU and the CAM-ICU Flowsheet are very similar tools. However, our previous study, despite an excellent correlation (kappa: 0.96) between these tools [9], showed that they were not identical, so we decided not to add the Guenther's study in this meta-analysis.

A Canadian group developed and validated the ICDSC [20] motivated by the same challenge: diagnosing delirium in critically ill and mechanically ventilated patients.

The ICDSC checklist is an eight-item screening tool (one point for each item) that is based on DSM criteria and applied to data that can be collected through medical records or to information obtained from the multidisciplinary team.

Bergeron *et al. *developed and validated the ICDSC in a mixed ICU [20]. All of the information used to complete the scale was collected from the patient, the primary nurses' evaluation and the chart in the previous 24 hours. With a cutoff score of four points, they showed a sensitivity of 99% and a specificity of 64%. Similar results were found by our group [9]. However, we observed that the sensitivity of the ICDSC was not consistently high in all studies, and that the pooled value for sensitivity in this meta-analysis was 74%. These results suggest that this tool does not appear to be as accurate as the CAM-ICU for screening purposes. George *et al.*, using a different threshold for positivity (3 rather than 4), showed a higher sensitivity (from 75% to 90%) and, consequently, improved screening characteristics of this tool [21]. However, these changes in the cutoff decreased the specificity of the ICDSC, which was already lower than that observed for the CAM-ICU. The pooled value for specificity of the ICDSC in this meta-analysis was 82%.

Additionally, a recent study by Tomasi *et al. *suggested that the CAM-ICU is a better predictor of outcomes than the ICDSC, which is likely related to the high rate of false positives with the ICDSC [26]. At least two characteristics of the ICDSC might explain its lower sensitivity and specificity. First, the information is collected from the previous 24 hours. Delirium is characterized by its fluctuation, with the possibility of resolution over a long period of evaluation. Additionally, the evaluation of inattention ("easily distracted by external stimuli" [20]), for example, may hinder an effective response by the evaluator.

Despite the limitations described above, the inter-rater reliability of the ICDSC appears to be good. George *et al. *[21] reported an inter-rater agreement of 0.947 (95% confidence interval, 0.870 to 0.979), and in the study by Bergeron *et al. *[20], the calculated alpha value was between 0.71 and 0.79.

Interestingly, both tools have worse sensitivity when patients with hypoactive delirium are tested. This issue is relevant because this subtype of delirium is the most prevalent [27]. A lower prevalence of delusions and perceptual disturbances in hypoactive delirium does not appear to explain these findings [28].

Despite the observation that no studies compared the accuracy of both tools in ventilated versus non-ventilated patients, most studies included these two types of patients.

Both tools are important in the care of the critically ill patients, each one with features that allow its use at different times or together. The CAM-ICU, to be quite specific, seems to be the ideal tool for the diagnosis of delirium in critically ill patients. In turn, the ICDSC, by its features not dichotomous, allows the diagnosis of subsyndromal delirium, which has potential prognostic implications [29] and can identify patients with potential therapeutic benefit [30].

Our findings should be understood in the context of some limitations. First, studies published in non-English languages were excluded. Unfortunately, a substantial part of the core information was not available from these studies precluding its use in the meta-analysis. However, as described above, the accuracy of the CAM-ICU appears to be consistent with the results of some of these studies. Second, this study cannot explain the findings with different accuracies of these tools in subgroups of patients (ventilated and nonventilated, RASS < 0, subtypes of delirium), but likely, this is a limitation of the tools. Additionally, the use of the CAM-ICU in patients with non-invasive ventilation has an excellent accuracy; however, its data are limited to a single study involving a small number of observations. This reflects the need for studies to evaluate specific groups of patients.

## Conclusions

The present meta-analysis demonstrates that the CAM-ICU is an excellent tool for the detection of delirium in critically ill ICU patients regardless of the subgroup of patients evaluated. Despite having a good performance, the ICDSC presents lower sensitivity and specificity as compared to CAM-ICU. The available data suggest that both CAM-ICU and the ICDSC can be used as a screening tool for the diagnosis of delirium in critically ill patients.

## Key messages

• The CAM-ICU and the ICDSC are the most studied tools for the diagnosis of delirium in critically ill patients.

• The CAM-ICU and the ICDSC are good screening tools for delirium in ICU patients.

• The CAM-ICU is an excellent diagnostic tool for delirium in critically ill ICU patients

## Abbreviations

AUC: area under the summary receiver operating characteristic curve; CAM-ICU: Confusion Assessment Method for the Intensive Care Unit; DSM-IV: Diagnostic and Statistical Manual of Mental Disorders; ICDSC: Intensive Care Delirium Screening Checklist; ICU: Intensive Care unit; RASS: Richmond Agitation Sedation Scale; SROC: summary receiver operating curve characteristic.

## Competing interests

There are no conflicts of interest related to this investigation to disclose.

## Authors' contributions

DGF and JIFS conceived the study. DGF and RAC performed the database searches. DGF undertook the statistical analysis. DGF, JIFS and LCQ contributed to the writing of the first draft of the manuscript. All of the authors contributed to and have approved the final manuscript.

## Supplementary Material

Additional file 1 **Figure S1**. Forest plot of the pooled values of sensitivity and specificity of the CAM-ICU (included pCAM-ICU). Figure S2. Summary receiver operating characteristics (SROC) obtained from the evaluation studies of the CAM-ICU (included pCAM-ICU). Click here for file

Additional file 2 **Table S1**. Main characteristics of the included studies (evaluation of the CAM-ICU). Included data from all evaluators. Click here for file
