# Supplementary material for: The confusion assessment method for the intensive care unit (CAM-ICU) and intensive care delirium screening checklist (ICDSC) for the diagnosis of delirium: a systematic review and meta-analysis of clinical studies
Source: Crit Care. 2012 Jul 3;16(4):R115. doi: 10.1186/cc11407 (PMC3580690; doi:10.1186/cc11407)
Supplement: Additional file 1 — Figure S1. Forest plot of the pooled values of sensitivity and specificity of the CAM-ICU (included pCAM-ICU). Figure S2. Summary receiver operating characteristics (SROC) obtained from the evaluation studies of the CAM-ICU (included pCAM-ICU). [file cc11407-S1.DOC]

**Additional file 1**

**Figure S1. Forest plot of the pooled values of sensitivity and specificity of the CAM-ICU (included pCAM-ICU).**

**Figure S2. Summary receiver operating characteristics (SROC) obtained from the evaluation studies of the CAM-ICU (included pCAM-ICU).**
