# Supplementary material for: The confusion assessment method for the intensive care unit (CAM-ICU) and intensive care delirium screening checklist (ICDSC) for the diagnosis of delirium: a systematic review and meta-analysis of clinical studies
Source: Crit Care. 2012 Jul 3;16(4):R115. doi: 10.1186/cc11407 (PMC3580690; doi:10.1186/cc11407)
Supplement: Additional file 2 — Table S1. Main characteristics of the included studies (evaluation of the CAM-ICU). Included data from all evaluators. [file cc11407-S2.DOC]

**Additional file 2**

**Table S1. Main characteristics of the included studies (evaluation of the CAM-ICU)**

Included data from all evaluators

| **Author** | **N** | **Year** | **ICU** | **Language** | **Delirium**  N (%) | **Sensitivity** | **Specificity** | **APACHE II** | **QUADAS** |
| --- | --- | --- | --- | --- | --- | --- | --- | --- | --- |
| **Ely**  **Nurse 1**  **Nurse 2**  **Intensivist** | 38  37  38  26 | 2001 | Medical/  coronary | English | 33 (87)^1^ | 95 (77-100)  96 (78-100)  100 (80-100) | 93 (68-100)  93 (68-100)  89 (51-100) | 17.1 ± 8.7^#^ | 13 |
| **Ely**  **Nurse 1**  **Nurse 2** | 96  91  92 | 2001 | Medical / coronary | English | (25.2)^2^ | 100 (90-100)  93 (82-99) | 98 (91-100)  100 (93-100) | 23 (18-29)^*^ | 13 |
| **Lin**  **Assessor 1**  **Assessor 2** | 102 | 2004 | Medical | Chinese | 22 (22.4) | 91  95 | 98  98 | NR | 13 |
| **Van Eijk** | 126 | 2009 | General | English | 43 (34) | 64 (49-77) | 88 (79-93) | 20. 9 ± 7.5^#^ | 14 |
| **Luetz** | 156 | 2010 | Surgical | German | 63 (40) | 79 | 97 | 16 (13-19)^*^ | 14 |
| **Heo**  **Nurse 1**  **Nurse 2** | 22 | 2011 | Medical | Korean | 16 (72.7) | 89.8  77.4 | 72.4  75.8 | 25.5 (9 – 39)^*^ | 13 |
| **Van Eijk** | 181 | 2011 |  | English Dutch | 80 (28.3) | 46.7 (35.1 – 58.6) | 98.1 (93.4 – 99.8) | 18.6 ± 7.5^#^ | 13 |
| **Gusmao-Flores** | 119 | 2011 | Medical/  surgical | Portuguese | 46 (38.6) | 72.5 (55.9 – 84.9) | 96.2 (88.5 – 99.0) | 15 ± 6^#^ | 14 |
| **Mitasova** | 129 | 2012 | Stroke | Czech | 55 (42.6)^1^ | 76 (54.9 – 90.6) | 98.1 (93.2 – 99.8) | NR | 14 |

^1^During hospitalization, ^2^% of daily evaluations, NR: not reported, ^#^mean, ^*^median.
